# Disseminated *Nocardia ignorata* Infection with Splenic and Brain Involvement in Patient with Large B-Cell Lymphoma

**DOI:** 10.3201/eid3201.251546

**Published:** 2026-01

**Authors:** Sherif Elbaz, Mahmoud Ismail, Seth Glassman, Asmaa Badr, Eric John Dove

**Affiliations:** University at Buffalo, Buffalo, New York, USA (S. Elbaz, M. Ismail, S. Glassman, E.J. Dove); The Ohio State University, Columbus, Ohio, USA (A. Badr)

**Keywords:** Nocardia ignorata, bacteria, bacterial infections, disseminated nocardiosis, spleen, brain, lymphoma, United States

## Abstract

A 79-year-old man in the United States with large B-cell lymphoma and chronic obstructive pulmonary disease had disseminated *Nocardia ignorata* infection involving the brain and spleen. Despite antimicrobial therapy, he died from complications. This rare manifestation highlights the need to consider *Nocardia* in immunocompromised patients with central nervous system and abdominal lesions.

Nocardia are filamentous, gram-positive, aerobic bacteria that infect immunocompromised hosts, causing pulmonary, cutaneous, or central nervous system disease (*1*–*4*). Dissemination to abdominal organs is rare (*5*). Among published cases, splenic involvement accounts for <10% of abdominal nocardiosis (*5*). We report disseminated *N. ignorata* infection with concurrent splenic and brain involvement in a patient in the United States who had large B-cell lymphoma.

## The Study

**Figure 1:**
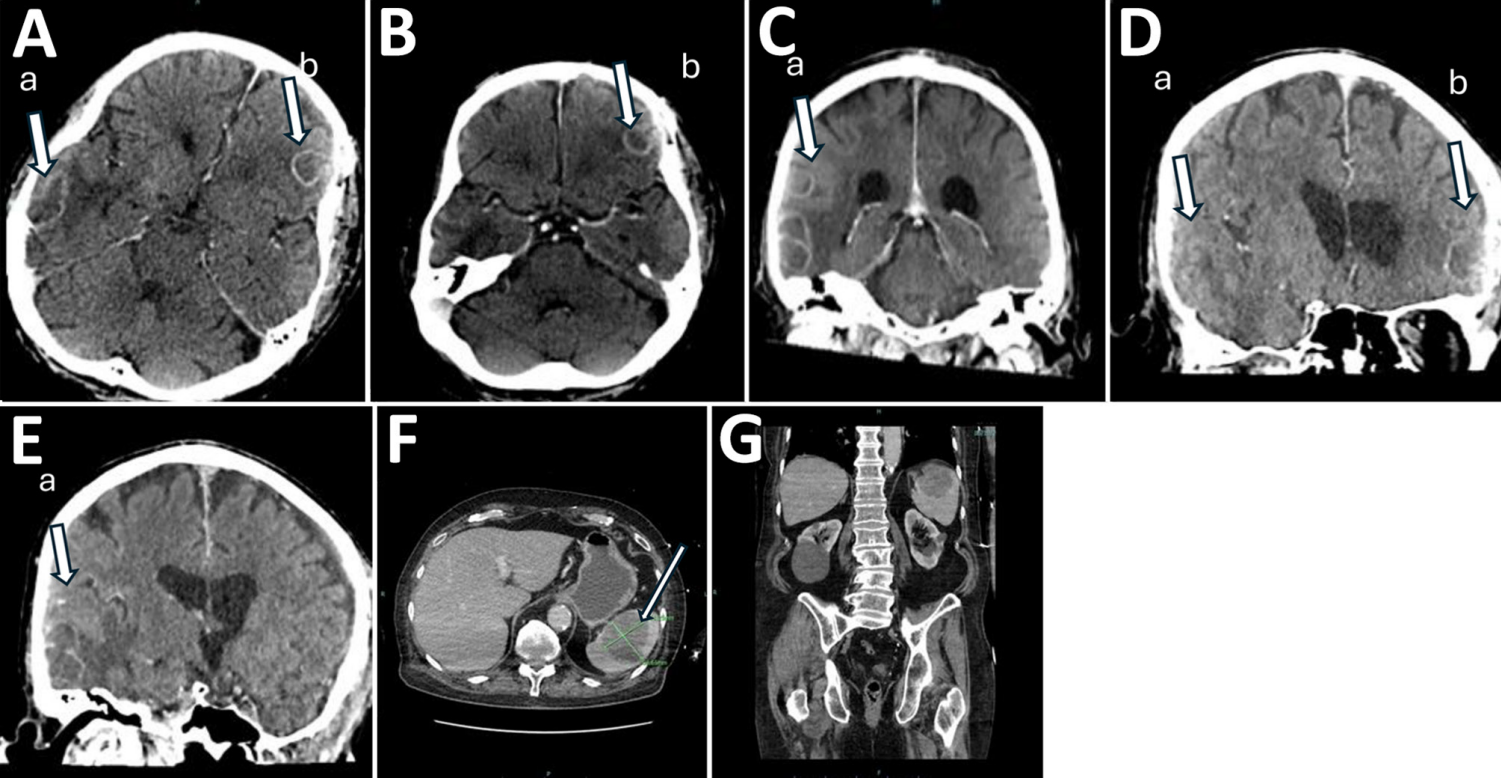
Computed tomography (CT) images of brain, abdomen, and spine of 79-year-old man with chronic obstructive pulmonary disease and large B-cell lymphoma who had disseminated *Nocardia ignorata* infection, United States. A–E) Brain CT with and without contrast showing multiple regular peripheral enhancing brain lesions (indicated by arrow and lowercase letters a and b) measuring 2.2 cm at the left frontal lobe (A) and 3.8 cm at the right (B); mildly lobulated at the right posterior lateral temporal lobe (C); and moderate edematous mass (D), resulting in mild brain herniation (E). F, G) CT images of abdomen (F) and pelvis (G). An indeterminate complex lesion measuring 6.1 cm is seen in the spleen (F, arrow).

A 79-year-old man with chronic obstructive pulmonary disease and large B-cell lymphoma had a new-onset seizure, left facial droop, and slurred speech. Computed tomography of the brain revealed multiple enhancing lesions with surrounding edema and mild herniation (Figure 1, panels A–E). Computed tomography of the abdominal pelvis (Figure 1, panel F, G) and chest showed a pulmonary nodule, a right renal lesion, and a 6.1-cm splenic mass.

**Figure 2:**
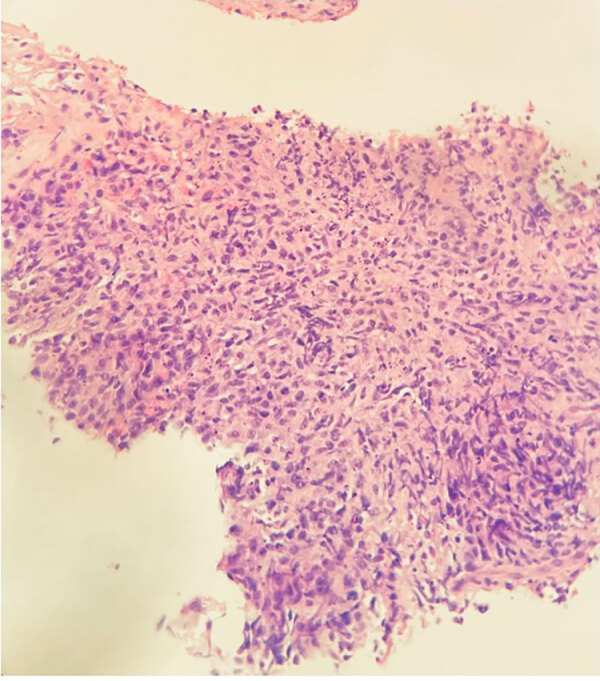
Ultrasound-guided biopsy image of spleen of 79-year-old man with chronic obstructive pulmonary disease and large B-cell lymphoma who had disseminated *Nocardia ignorata* infection, United States. Tissue fragment shows extensive necrosis with a small focus of large B-cell lymphoma. Neoplastic cells stain positively for CD20 antibody, paired box protein 5, and B‑cell lymphoma 2, with 20% expression of myelocytomatosis oncogene, corroborating the diagnosis of disseminated *Nocardia ignorata* infection. Mindbomb‑1 antibody is positive in 90% of the cells. Acid-fast, Fite–Faraco, Gomori methenamine silver, and periodic acid–Schiff special stains do not demonstrate identifiable microorganisms or microbiota. Original magnification × 20.

**Figure 3:**
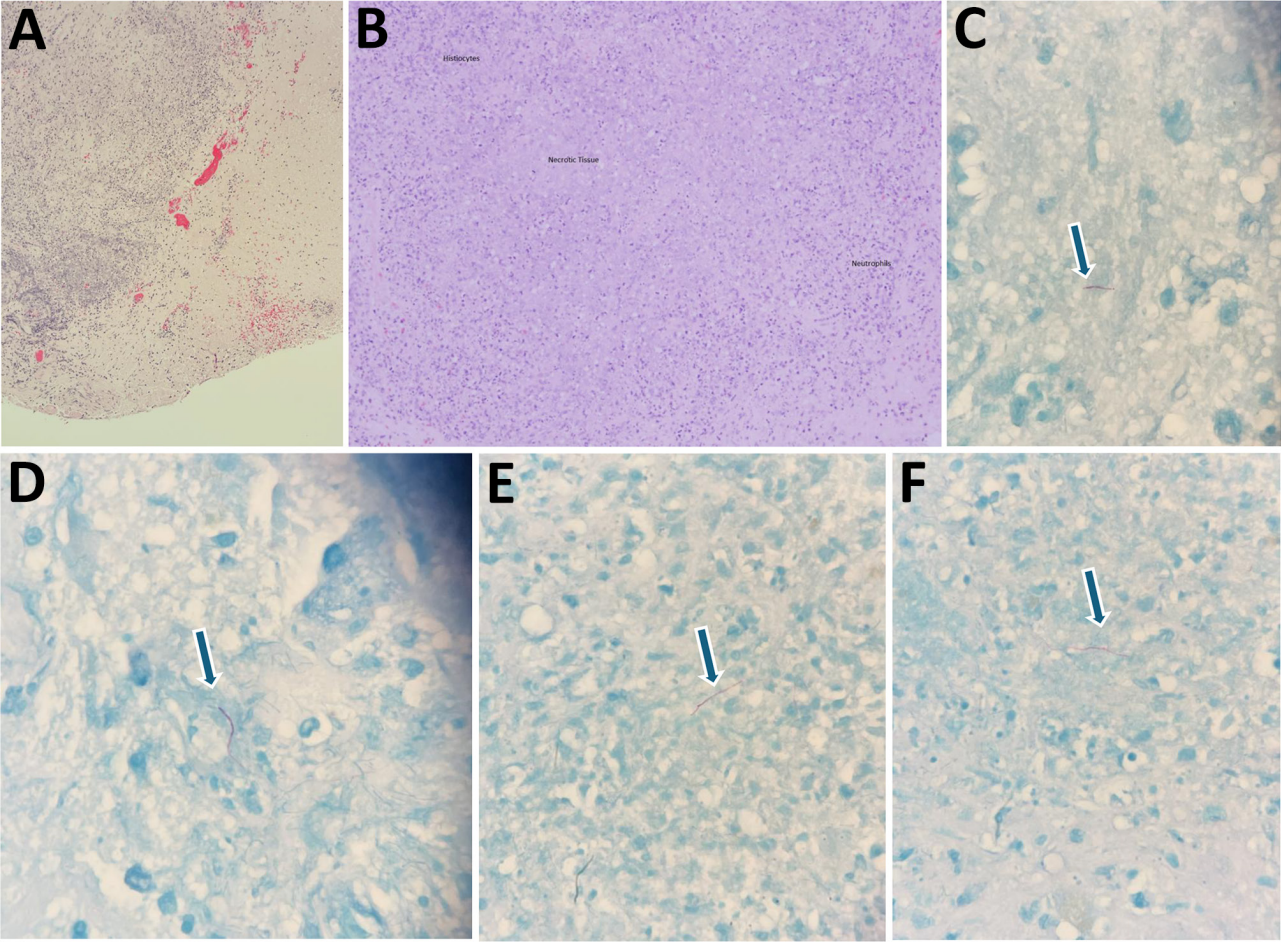
Brain tissue images from 79-year-old man with chronic obstructive pulmonary disease and large B-cell lymphoma who had disseminated *Nocardia ignorata* infection, United States. A, B) Parenchyma with identifiable granulation tissue, neutrophils, and histocytes and background hemorrhage and necrosis are seen. Hematoxylin and eosin stain; original magnification ×10. C–F) Thin, filamentous, partially acid-fast rods with a beaded appearance within the necrotic tissue background are visible. Branching within visualized organisms was not identified. Arrows indicate *N. ignorata* bacteria. Fite–Faraco stain; original magnification × 100.

Biopsy of the spleen demonstrated an extensively necrotic B-lymphocyte antigen cluster of differentiation 20–positive large B-cell lymphoma and limited viable tissue, explaining the negative primary stains we obtained (Figure 2). Cultures of splenic aspiration and brain biopsy grew filamentous branching *N.* *ignorata* (Figure 3). We used matrix-assisted laser desorption/ionization time-of-flight mass spectrometry for species-level identification. Blood culture results were negative. We used blood agar and chocolate agar for isolation. We extended the incubation period to accommodate slow growth.

We started the patient on antimicrobial therapy (intravenous imipenem and oral trimethoprim/sulfamethoxazole and linezolid) before susceptibility results were available. The patient’s hospital course was complicated by upper gastrointestinal bleeding. He was transitioned to comfort care and subsequently died.

## Conclusions

Disseminated nocardiosis frequently affects the lungs, brain, or skin, but splenic involvement is rare (*2*,*3*,*5*). Immunosuppression from malignancy predisposes patients to opportunistic infections such as those caused by *Nocardia* (*2*,*3*,*6*). This case highlights the diagnostic challenge posed by nonspecific clinical findings and the need for microbiologic confirmation, given that *Nocardia* species exhibit variable antimicrobial drug susceptibility (*1*,*3*). The observed pattern of simultaneous brain and splenic involvement in the setting of lymphoma underscores the need to consider the emergence of this disease in immunocompromised patients. Our findings also support early inclusion of nocardiosis in the differential diagnosis for patients with concurrent central nervous system and visceral lesions (*2*,*3*,*5*,*7*).
